# Author Correction: Widespread extinction debts and colonization credits in United States breeding bird communities

**DOI:** 10.1038/s41559-022-01912-x

**Published:** 2022-10-10

**Authors:** Yacob Haddou, Rebecca Mancy, Jason Matthiopoulos, Sofie Spatharis, Davide M. Dominoni

**Affiliations:** 1 Institute of Biodiversity, Animal Health and Comparative Medicine, University of Glasgow, Glasgow, UK; 2 Social and Public Health Sciences Unit, University of Glasgow, Glasgow, UK; 3 School of Life Sciences, University of Glasgow, Glasgow, UK

**Keywords:** Biodiversity, Ecological modelling

Correction to: *Nature Ecology & Evolution* 10.1038/s41559-021-01653-3, published online 10 February 2022.

In the version of this article initially published, there were errors in equations and notations in the Methods “Model development” subsection which arose during manuscript preparation; the errors affect presentation of the study but not the analysis, results, or code provided with the article. Clarifications to text and equations follow.

In Equation (1), “*N*” replaces “Normal”; in Equations (2), (3), (7) and in text directly below Equations (3), (5) and (7), “*y*_*s,i,z*_” now replaces “Δ*x*_*s,t*1_, *t*_2_.” In the two paragraphs below Equation (2), “*t*_2_ = 2016” and “*t*_1_ = 2001” now replace “2016” and “2001” in five instances. Further, Equations (5)–(7) have been revised as follows:

$$\begin{array}{ll}f\left( {x_{s,t}} \right) = {{{\mathrm{exp}}}} & \left( {\beta _0 + \mathop {\sum }\limits_{i = 1}^{I = 5} \beta _{1,i} x_{s,i,t} + \mathop {\sum }\limits_{i = 1}^{I = 5} \mathop {\sum }\limits_{k = i}^{K = 5} \beta _{2,i,k}x_{s,i,t}x_{k,s,t}}\right. \\ & \quad \quad \left. {+ \mathop {\sum }\limits_{i = 1}^{I = 5} \mathop {\sum }\limits_{k = 1, k \neq i}^{K = 5} \beta _{3,i,k}x_{s,i,t}x_{k,s,t}} \right)\end{array}\ {\rm{Revised}}\ {\rm{Eq}}.\ (5)$$

$$\begin{array}{ll}f\left( {x_{s,t}} \right) \\ = exp\left( {\beta _0 + \mathop {\sum }\limits_{i = 1}^{I = 5} \mathop {\sum }\limits_{j = 1}^{J = 2} \beta _{0,i,j,}x_{i,s,t}^j + \mathop {\sum }\limits_{i = 1}^{I = 5} \mathop {\sum }\limits_{k = i + 1}^{K = 6} \beta _{1,i,k}x_{i,s,t}x_{k,s,t}} \right) {\mathrm{Original}}\ {\rm{Eq}}.\ (5)\end{array}$$

$$y_{s,i,z} = \left\{ {\begin{array}{*{20}{l}} {y_{s,i,1} = \left| {\Delta x_{s,i}} \right|,} \hfill & {y_{s,i,2} = 0,} \hfill & {{{{\mathrm{if}}}}\,\Delta x_{s,i} < 0} \hfill \\ {y_{s,i,1} = 0,} \hfill & {y_{s,i,2} = \Delta x_{s,i}} \hfill & {{{{\mathrm{otherwise}}}}} \hfill \end{array}} \right.\ {\rm{Revised}}\ {\rm{Eq}}.\ (6)$$

$$x_{i,s,} = \left\{ {\begin{array}{*{20}{l}} {x_{1,i,s} = \left| {\Delta x_{i,s}} \right|,} \hfill & {x_{2,i,s} = 0,} \hfill & {if\,\Delta x_{i,s} < 0} \hfill \\ {x_{1,i,s} = 0,} \hfill & {x_{2,i,s} = \Delta x_{i,s},} \hfill & {otherwise} \hfill \end{array}} \right.\ {\rm{Original}}\ {\rm{Eq}}.\ (6)$$

$$\omega \left( {y_{s,i,z};\gamma } \right) = {{{\mathrm{exp}}}}\left( {\mathop {\sum }\limits_{i = 1}^{I = 5} \mathop {\sum }\limits_{z = 1}^{Z = 2} - \gamma _{i,z} y_{s,i,z}} \right)\ {\rm{Revised}}\ {\rm{Eq}}.\ (7)$$

$$\omega \left( {\Delta x_{s,t_1,t_2};\gamma } \right) = exp\left( {\mathop {\sum }\limits_{i = 1}^{I = 5} - \gamma _{i,z}\Delta x_{z,s,i}} \right)\ {\rm{Original}}\ {\rm{Eq}}.\ (7)$$

All changes have been made in the HTML and PDF versions of the article.

