# Supplementary material for: Widespread extinction debts and colonization credits in United States breeding bird communities
Source: Nat Ecol Evol. 2022 Feb 10;6(3):324–31. doi: 10.1038/s41559-021-01653-3 (PMC8913367; doi:10.1038/s41559-021-01653-3)
Supplement: Supplementary file 1 — Supplementary Figs. 1–4 and Tables 1–3. [file 41559_2021_1653_MOESM1_ESM.pdf]

nature portfolio

https://doi.org/10.1038/s41559-021-01653-3

Supplementary information

Widespread extinction debts and colonization credits in United States breeding bird communities

In the format provided by the authors and unedited

Supplementary Information for:

Widespread extinction debts and colonisation credits in United States breeding bird communities

List of Supplementary Information:

Supplementary Figures 1-4

Supplementary Tables 1-3

Supplementary figures

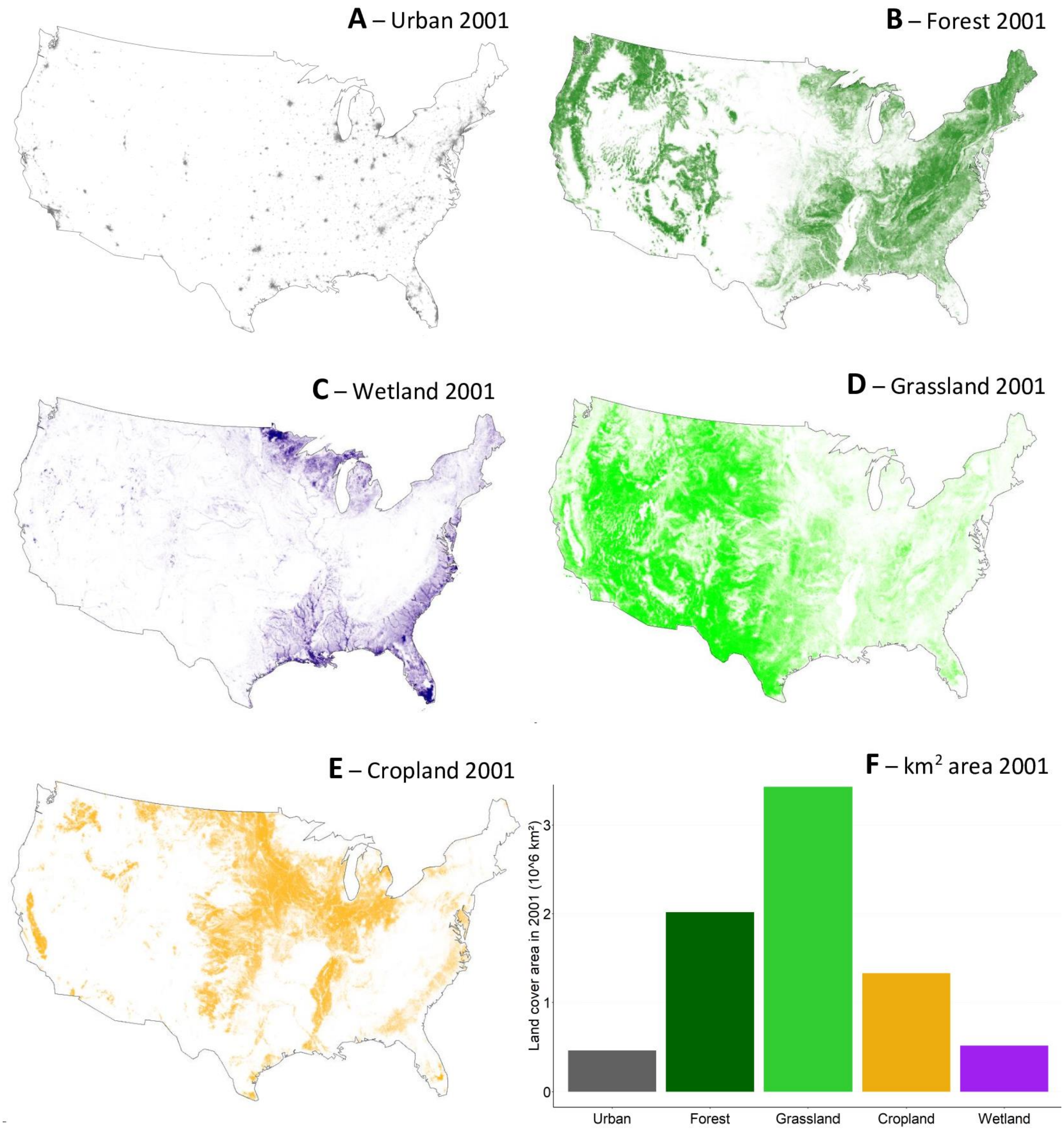

Supplementary Figure 1. Distributions of environmental covariates across the USA in 2001.

Maps of the contiguous USA states showing the 2001 spatial distribution (A-E) and area (F) of each land cover included in the analysis. Land cover variables were produced as aggregate: Urban (panel A) [an aggregate of the Developed-Open Space (sub-class 21), Developed-Low Intensity (22), Developed-Medium Intensity (23), Developed-High Intensity classes]; Forest (panel B) [an aggregate of the Deciduous Forest (41), Evergreen Forest (42), Mixed Forest (43) classes]; Wetland (panel C) [an aggregate of the Woody Wetland (90) and Herbaceous Wetland (95) classes]; Grassland (panel D) [an aggregate of the Shrub (52), Grassland/Herbaceous (71), Pasture/Hay (81) classes] and Cropland (panel E) [cultivated Crops (82) sub class].

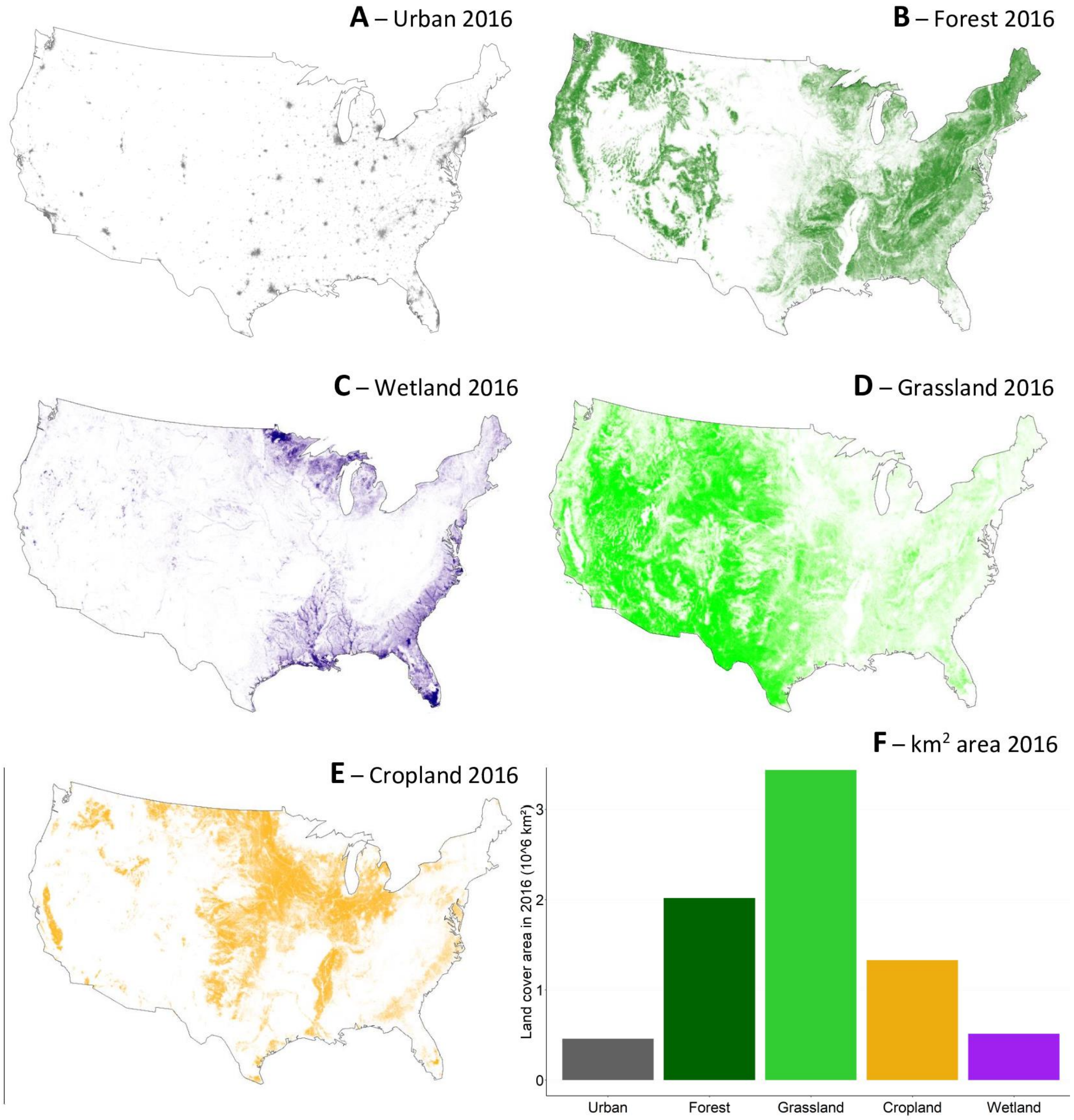

Supplementary Figure 2. Distributions of environmental covariates across the USA in 2016.

Maps of the contiguous USA states showing the 2016 spatial distribution (A-E) and area (F) of each land cover included in the analysis. Land cover variables were produced as aggregate: Urban (panel A) [an aggregate of the Developed-Open Space (sub-class 21), Developed-Low Intensity (22), Developed-Medium Intensity (23), Developed-High Intensity classes]; Forest (panel B) [an aggregate of the Deciduous Forest (41), Evergreen Forest (42), Mixed Forest (43) classes]; Wetland (panel C) [an aggregate of the Woody Wetland (90) and Herbaceous Wetland (95) classes]; Grassland (panel D) [an aggregate of the Shrub (52), Grassland/Herbaceous (71), Pasture/Hay (81) classes] and Cropland (panel E) [cultivated Crops (82) sub class].

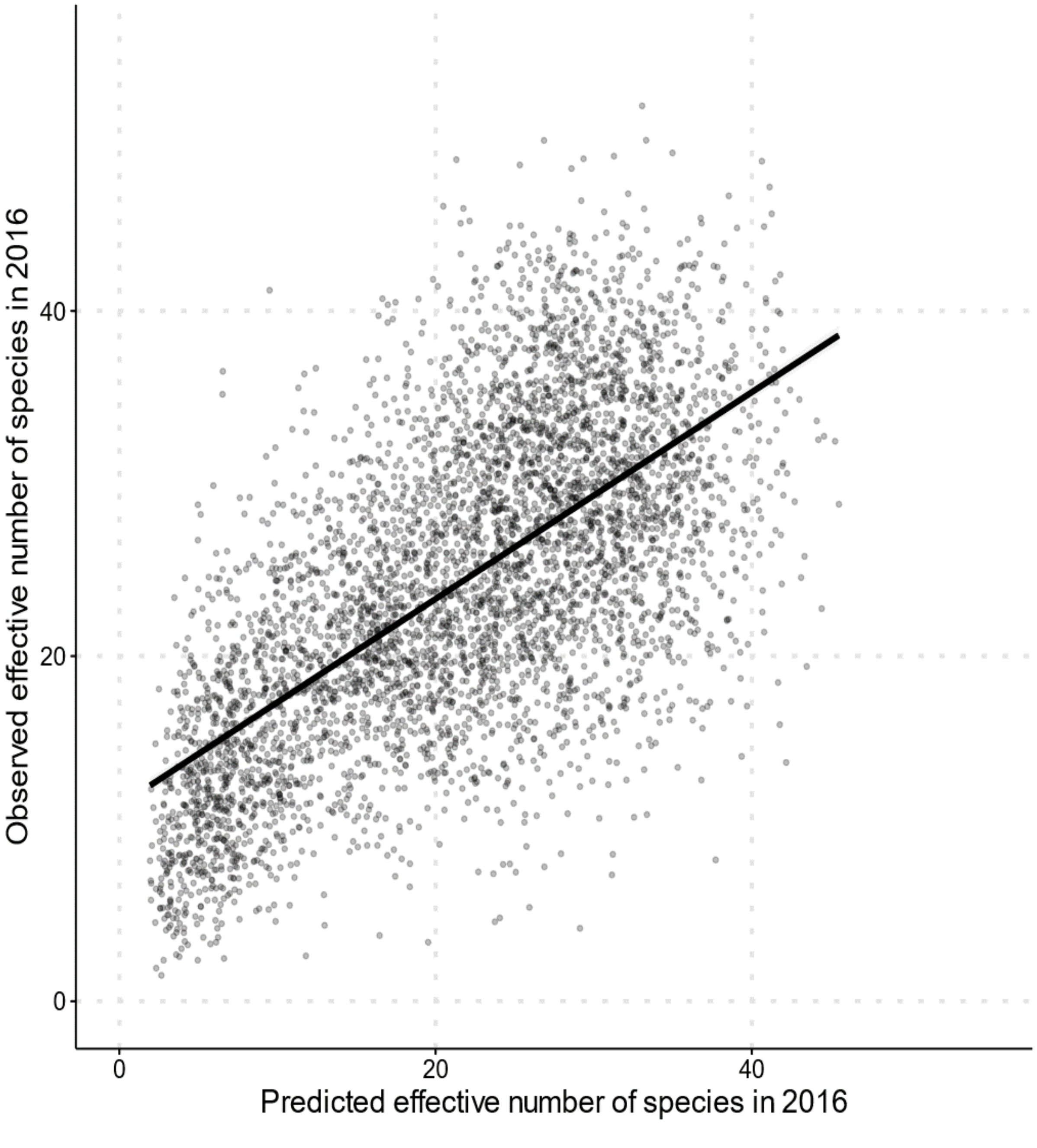

Supplementary Figure 3. Correlation between the observed effective number of species in 2016 and the model predicted effective number of species in 2016. Scatter plot of the observed effective number of species in 2016, across the 4800 analysed US bird communities (including all segments, not only 1-3-5 as per model fit), against the model predicted value of effective number of species. The model was able to explain 42% of the observed variation. The model-predicted effective number of species was significantly correlated to the observed effective number of species in 2016 (Pearson's $r = 0.65$, $df = 4798$, $p < 0.01$).

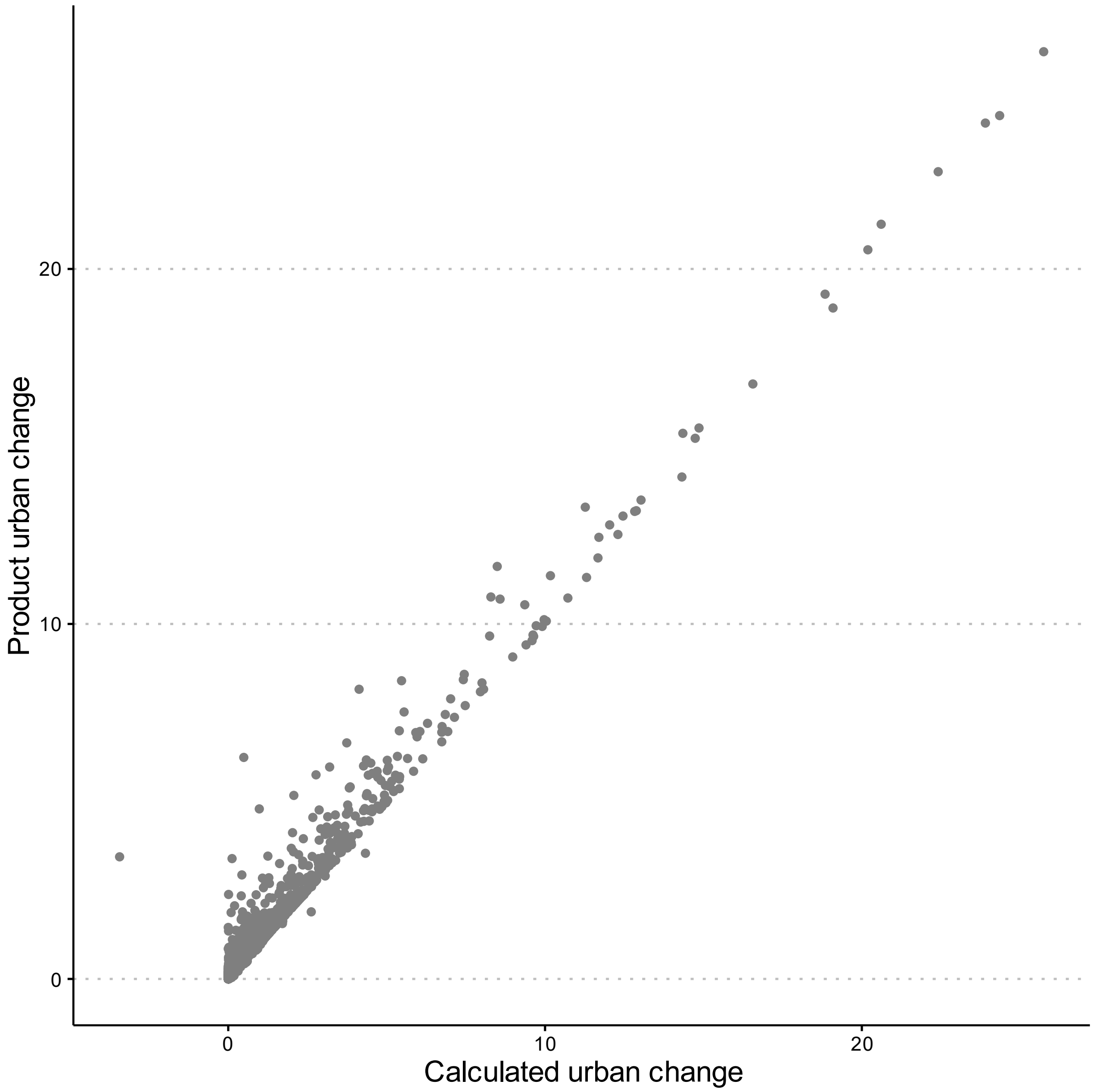

Supplementary Figure 4. Validation of land cover change data. On the x-axis we show the percentage point change in the urban land cover between the years 2016 and 2001, as calculated using the two independent USGS land cover maps produced for the two years. On the y-axis, we show the values recently published in the “change product” map provided by the USGS[40]. We used only the urban land cover for this validation because this is the only land cover directly comparable between the static and the product change map. The change product map does not include the directionality of change in its published data. However, because the urban land cover has seen only increase (i.e. unidirectional change) over the 15-year period used in our analyses, it can therefore be used for this validation.

Supplementary Tables

Supplementary Table 1. Descriptive statistics of the analysed variables.

Variable	Mean	Std. Dev	Median	Min	Max
Environmental covariates at t1(year 2001) and t2(year 2016) (numbers indicate proportions between 0 and 100)					
Urban t_1	8.23	8.84	5.94	0.00	97.11
Urban t_2	8.64	9.54	6.01	0.00	97.30
**Developed-openspace t_1**	3.56	3.41	3.06	0.00	44.06
**Developed-openspace t_2**	3.67	3.57	3.08	0.00	43.92
**Developed-low t_1**	1.46	3.07	0.52	0.00	38.41
**Developed-low t_2**	1.54	3.28	0.55	0.00	38.90
**Developed-medium t_1**	0.42	1.50	0.04	0.00	27.29
**Developed-medium t_2**	0.52	1.76	0.05	0.00	28.30
**Developed-medium t_1**	0.11	0.52	0.00	0.00	12.39
**Developed-medium t_2**	0.15	0.62	0.00	0.00	13.15
Forest t_1	30.08	29.93	22.73	0.00	99.92
Forest t_2	30.19	29.40	22.36	0.00	98.08
**Deciduous forest t_1**	16.82	22.67	4.62	0.00	93.34
**Deciduous forest t_2**	16.45	22.36	4.40	0.00	93.52
**Evergreen forest t_1**	11.31	20.76	0.93	0.00	98.90
**Evergreen forest t_2**	10.86	19.69	0.93	0.00	98.73
**Mixed forest t_1**	5.97	9.61	1.19	0.00	67.74
**Mixed forest t_2**	6.01	9.60	1.23	0.00	67.86
Wetland t_1	6.66	13.21	0.99	0	96.72
Wetland t_2	6.68	13.22	1.03	0	96.72
**Woody wetlands t_1**	5.75	11.78	0.55	0.00	94.98
**Woody wetlands t_2**	5.80	11.86	0.56	0.00	96.54
**Herbaceous wetlands t_1**	1.67	5.65	0.16	0.00	94.43
**Herbaceous wetlands t_2**	1.65	5.55	0.17	0.00	94.17
Grassland t_1	33.15	31.13	21.87	0	100.00
Grassland t_2	32.81	30.82	21.29	0	100.00
**Grassland herbaceous t_1**	9.58	19.88	0.88	0.00	99.87
**Grassland herbaceous t2**	9.84	19.77	1.22	0.00	99.87
**Shrubland t_1**	12.88	25.11	0.83	0.00	100.00
**Shrubland t_2**	13.14	24.91	1.09	0.00	100.00
**Pasture t_1**	9.13	13.79	2.78	0.00	84.32
**Pasture t_2**	8.50	12.96	2.59	0.00	83.26
Cropland t_1	19.30	28.22	1.86	0.00	95.83
Cropland t_2	19.94	28.42	2.57	0.00	95.82
Temp t_1	18.51	4.25	18.32	6.64	30.22
Temp t_2	18.65	3.97	18.44	6.74	32.08
**Barren land t_1**	0.42	2.58	0.02	0.00	58.56

**Barren land t_2**	0.41	2.48	0.03	0.00	60.92
**Water t_1**	1.41	3.91	0.13	0.00	48.25
**Water t_2**	1.41	3.85	0.13	0.00	48.32
Landscape heterogeneity t_1	3.79	1.76	3.57	1.00	10.71
Landscape heterogeneity t_2	3.89	1.82	3.68	1.00	10.72
Time of day t_2 (minutes after earliest survey overall)	329.2	175.91	327.00	0.00	860.00
Change in environmental covariates between t2 and t1, 2016 and 2001					
Delta urban	0.31	1.32	0.00	0.00	25.74
**Delta pos urban**	0.31	1.32	0	0	25.74
**Delta neg urban**	-	-	-	-	-
Delta forest	-0.61	4.09	0.00	-78.68	45.04
**Delta pos forest**	0.37	1.68	0.00	0	45.04
**Delta neg forest**	0.99	3.63	0.00	0	78.68
Delta wetland	0.019	0.45	0.00	-5.57	10.53
**Delta pos wetland**	0.06	0.38	0	0	10.53
**Delta neg wetland**	0.04	0.22	0	0	5.57
Delta grassland	-0.34	4.70	-0.10	-45.04	78.66
**Delta pos grassland**	0.87	3.59	0.00	0	78.66
**Delta neg grassland**	1.21	2.66	0.10	0	45.04
Delta cropland	0.63	2.28	0.00	-23.91	31.05
**Delta pos cropland**	0.78	2.06	0.00	0	31.05
**Delta neg cropland**	0.14	0.84	0.00	0	23.91
Biodiversity variables					
Effective no. species t_2	24.29	9.05	24.18	1.50	51.88
Effective no. species t_1	23.67	9.27	23.64	1.35	54.16
Delta Effective no. species	0.62	5.84	0.49	-32.04	27.84
Species richness t_2	43.02	12.74	44.00	4.00	87.00
Species richness t_1	43.15	13.20	44.00	2.00	89.00
Delta species richness	-0.12	7.70	0	-37.00	31.00

Supplementary Table 2. Summary of parameter estimates of the equilibrium and legacy model components. Parameters are subdivided according to the model sections as described in the model development section of the Supplementary Materials. Values of rhat indicate chain convergence (ideally these should approach 1), while the ess_bulk and ess_tail indicate the effective sample size, either at the bulk or tails, of the posterior distributions, with values above 400 being considered satisfactory.

Parameter	mean	median	SD	q5	q95	rhat	ess_bulk	ess_tail
Equilibrium model [Eq. 5]								
intercept	0.992	0.710	0.946	0.058	2.893	1.001	4544	2206
urban	0.618	0.498	0.503	0.048	1.633	1.000	3239	1919
forest	1.334	1.267	0.711	0.246	2.591	1.000	2275	1693
grass	1.089	0.998	0.676	0.149	2.312	1.000	3196	1728
crop	1.459	1.423	0.777	0.260	2.802	1.001	2557	1908
wet	1.642	1.594	0.725	0.514	2.903	1.000	1557	1098
urban^2	-0.390	-0.382	0.194	-0.721	-0.087	1.001	1637	1018
forest^2	-0.376	-0.361	0.198	-0.729	-0.072	1.000	1460	1538
grass^2	-0.587	-0.577	0.181	-0.897	-0.309	1.002	1368	1227
crop^2	-0.732	-0.722	0.257	-1.179	-0.318	1.001	1623	1668
wet^2	-0.314	-0.289	0.198	-0.669	-0.032	1.001	1181	997
urban * forest	0.152	0.155	0.071	0.030	0.262	1.001	2327	1974
urban * grass	0.101	0.096	0.526	-0.786	0.955	1.002	1419	2019
urban * crop	-0.304	-0.298	0.496	-1.124	0.496	1.001	2506	2698
forest * grass	0.297	0.301	0.363	-0.295	0.907	1.001	1453	2426
forest * crop	-0.111	-0.106	0.342	-0.679	0.435	1.004	1998	2618
grass * crop	0.341	0.334	0.396	-0.310	0.987	1.000	1688	2440
wet * urban	0.625	0.631	0.466	-0.156	1.391	1.001	2574	2926
wet * forest	-0.794	-0.800	0.346	-1.353	-0.230	1.002	2189	2418
wet* grass	0.866	0.872	0.447	0.131	1.576	1.002	1843	2306
wet * crop	-0.918	-0.920	0.330	-1.460	-0.379	1.001	2399	2897
urban^2 * forest	-0.187	-0.186	0.054	-0.278	-0.097	1.002	2366	2951
urban^2 * grass	0.058	0.058	0.060	-0.040	0.157	1.000	2667	3062
urban^2 * wet	-0.089	-0.088	0.087	-0.231	0.058	1.001	2802	2954
urban^2 * crop	-0.120	-0.120	0.099	-0.284	0.040	1.000	2703	2640
forest^2 * urban	0.110	0.110	0.052	0.024	0.195	1.003	1650	2724

forest^2 * crop	0.085	0.085	0.056	-0.007	0.178	1.001	2189	3014
forest^2 * grass	0.051	0.050	0.049	-0.028	0.134	1.000	1782	2590
forest^2 * wet	-0.026	-0.026	0.070	-0.143	0.089	1.001	2234	2711
grass^2 * urban	-0.038	-0.038	0.103	-0.206	0.135	1.001	1494	2459
grass^2 * forest	0.017	0.018	0.063	-0.091	0.121	1.002	1635	2488
grass^2 * crop	0.027	0.027	0.060	-0.069	0.127	1.000	1742	2550
grass^2 * wet	-0.046	-0.046	0.079	-0.173	0.082	1.000	1925	2469
crop^2 * urban	0.180	0.178	0.110	0.002	0.368	1.002	3037	3184
crop^2 * forest	0.050	0.050	0.061	-0.051	0.150	1.004	2194	2528
crop^2 * grass	-0.092	-0.093	0.063	-0.194	0.013	1.002	1820	2530
crop^2 * wet	0.136	0.136	0.068	0.022	0.247	1.001	2481	3363
wet^2 * urban	-0.098	-0.097	0.102	-0.269	0.069	1.001	3311	2922
wet^2 * forest	0.218	0.218	0.059	0.122	0.314	1.002	2634	2525
wet^2 * grass	-0.205	-0.205	0.067	-0.314	-0.094	1.004	2252	2903
wet^2 * crop	0.189	0.190	0.059	0.091	0.284	1.001	2791	3057
Temporal legacy model [Eq. 7]								
γ pos urban	0.490	0.474	0.291	0.045	0.949	1.002	8017	2720
γ pos forest	0.397	0.344	0.277	0.032	0.909	1.001	5538	2592
γ pos grass	0.194	0.136	0.186	0.013	0.603	1.002	3451	2489
γ pos crop	0.462	0.446	0.276	0.055	0.926	1.003	6575	2288
γ pos wet	0.385	0.324	0.291	0.022	0.921	1.001	6675	2290
γ neg forest	0.193	0.139	0.180	0.013	0.582	1.001	3104	2268
γ neg grass	0.515	0.520	0.276	0.075	0.945	1.002	5292	2942
γ neg crop	0.541	0.555	0.279	0.075	0.961	1.000	5068	2538
γ neg wet	0.448	0.429	0.282	0.039	0.930	1.001	5842	2367
Static covariates								
time of day	0.005	0.005	0.000	0.004	0.006	1.001	3680	2793
temperature	1.515	1.513	0.252	1.105	1.928	1.004	946	1066
temperature^2	-0.040	-0.040	0.007	-0.051	-0.029	1.004	980	1055
landscape heterogeneity	2.990	2.994	0.579	2.026	3.925	1.001	3749	2838
SD observer random effect	3.866	3.863	0.154	3.622	4.119	1.003	2204	2675

Supplementary Table 3. Parameter summary of the linear model describing the effect of different types and directionalities of land cover change on the magnitude of our predicted extinction debts and colonisation credits. Coefficient estimates and uncertainty are also presented in Fig. 4 of the main text. Note that the means and standard deviations presented are the result of uncertainty propagation by fitting a GLM to 1000 response variable sets based on predictions from posterior draws and subsequently sampling 1000 times from each GLM fit parameter distributions before computing summary statistics.

**Parameter**	**mean**	**sd**	**2.5%**	**97.5%**
**Intercept**	0.046	0.010	0.026	0.066
**Urban gain**	-0.115	0.020	-0.156	-0.075
**Forest gain**	-0.018	0.021	-0.059	0.024
**Forest loss**	0.085	0.003	0.08	0.09
**Grassland gain**	0.006	0.020	-0.034	0.046
**Grassland loss**	0.039	0.019	0.002	0.077
**Cropland gain**	-0.059	0.023	-0.104	-0.149
**Cropland loss**	-0.005	0.022	-0.048	0.038
**Wetland gain**	0.036	0.025	-0.013	0.085
**Wetland loss**	-0.051	0.025	-0.101	-0.001
