# Supplementary material for: Widespread extinction debts and colonization credits in United States breeding bird communities
Source: Nat Ecol Evol. 2022 Feb 10;6(3):324–31. doi: 10.1038/s41559-021-01653-3 (PMC8913367; doi:10.1038/s41559-021-01653-3)
Supplement: Supplementary file 2 — Reporting Summary [file 41559_2021_1653_MOESM2_ESM.pdf]

nature portfolio

Corresponding author(s): Davide M Dominoni

Last updated by author(s): Dec 6, 2021

Reporting Summary

Nature Portfolio wishes to improve the reproducibility of the work that we publish. This form provides structure for consistency and transparency in reporting. For further information on Nature Portfolio policies, see our Editorial Policies and the Editorial Policy Checklist.

Statistics

For all statistical analyses, confirm that the following items are present in the figure legend, table legend, main text, or Methods section.

n/a	Confirmed	
☐	☒	The exact sample size (*n*) for each experimental group/condition, given as a discrete number and unit of measurement
☐	☒	A statement on whether measurements were taken from distinct samples or whether the same sample was measured repeatedly
☐	☒	The statistical test(s) used AND whether they are one- or two-sided<br>*Only common tests should be described solely by name; describe more complex techniques in the Methods section.*
☐	☒	A description of all covariates tested
☐	☒	A description of any assumptions or corrections, such as tests of normality and adjustment for multiple comparisons
☐	☒	A full description of the statistical parameters including central tendency (e.g. means) or other basic estimates (e.g. regression coefficient) AND variation (e.g. standard deviation) or associated estimates of uncertainty (e.g. confidence intervals)
☐	☒	For null hypothesis testing, the test statistic (e.g. *F*, *t*, *r*) with confidence intervals, effect sizes, degrees of freedom and *P* value noted<br>*Give P values as exact values whenever suitable.*
☐	☒	For Bayesian analysis, information on the choice of priors and Markov chain Monte Carlo settings
☐	☒	For hierarchical and complex designs, identification of the appropriate level for tests and full reporting of outcomes
☒	☐	Estimates of effect sizes (e.g. Cohen's *d*, Pearson's *r*), indicating how they were calculated

Our web collection on statistics for biologists contains articles on many of the points above.

Software and code

Policy information about availability of computer code

Data collection	RAll data utilised in the analysis is open access. Data on bird abundances can be accessed at: https://www.pwrc.usgs.gov/BBS/RawData/. Data on the land cover and temperature covariates can be accessed at: https://www.mrlc.gov/ (land cover) and https://prism.oregonstate.edu/ (temperature). BBS routes were sourced from https://databasin.org/datasets/02fe0ebbb 1b04111b0ba1579b89b7420/
Data analysis	Reproducible R code and processed datasets are available from https://github.com/valiriel/USBBS_Biodiversity_LandCover_Delays.

For manuscripts utilizing custom algorithms or software that are central to the research but not yet described in published literature, software must be made available to editors and reviewers. We strongly encourage code deposition in a community repository (e.g. GitHub). See the Nature Portfolio guidelines for submitting code & software for further information.

Data

Policy information about availability of data

All manuscripts must include a data availability statement. This statement should provide the following information, where applicable:

- Accession codes, unique identifiers, or web links for publicly available datasets
- A description of any restrictions on data availability
- For clinical datasets or third party data, please ensure that the statement adheres to our policy

All data utilised in the analysis is open access. Data on bird abundances can be accessed at: https://www.pwrc.usgs.gov/BBS/RawData/. Data on the land cover and temperature covariates can be accessed at: https://www.mrlc.gov/ (land cover) and https://prism.oregonstate.edu/ (temperature). BBS routes were sourced from https://databasin.org/datasets/02fe0ebbb 1b04111b0ba1579b89b7420/

Field-specific reporting

Please select the one below that is the best fit for your research. If you are not sure, read the appropriate sections before making your selection.

☐ Life sciences ☐ Behavioural & social sciences ☒ Ecological, evolutionary & environmental sciences

For a reference copy of the document with all sections, see nature.com/documents/nr-reporting-summary-flat.pdf

Ecological, evolutionary & environmental sciences study design

All studies must disclose on these points even when the disclosure is negative.

Study description	We used species richness data from the North American Breeding Bird Survey (BBS), comprising information on the abundance of 541 bird species across the contiguous USA. We also sourced high spatial resolution (30m2) land cover data from the National Land Cover Database CONUS products, as well as temperature data (mean across May and July) from the PRISM climate dataset. Using these datasets, we developed a generalized mixed effects model (GLMM) within a Bayesian framework describing the number of species in 2016 as a function of the weighted contribution of landscape composition in 2001 and 2016. This enabled us to explicitly quantify the importance of legacy effects in the response of bird communities to % changes in each of five major land cover classes (forest, grassland, cropland, wetland and urban area).
Research sample	We used the North American Breeding Bird Survey (BBS) dataset as our source of biodiversity data due to its long temporal coverage and spatial extent. The BBS is composed of bird species abundance records collected since 1966 from over 4000 survey routes across the countries of Mexico, USA and Canada.
Sampling strategy	Our final dataset included species richness and evenness data for 960 routes, each divided into five segments, giving a total of 2880 observational units (that we refer to as "segments").
Data collection	Data collection follows public access roads along non-linear transects that are 24.5 miles long (circa 39.2 Km) using a point count protocol whereby routes are surveyed every half-mile (800 m) for a total of 50 stops. At each stop, observers stand for three minutes and record the species and the abundance of every bird seen or heard within 400 meters of their location. The routes are surveyed by volunteers with experience in bird observation, and surveys are conducted during May and July to capture the peak breeding season.
Timing and spatial scale	To address our research questions, we selected the years 2001 and 2016 as our two timepoints. This 15-year timeframe was selected as a reasonable scale to explore biodiversity lags to land cover change and it also corresponded to the longest possible timespan for which land cover data products were available at high spatial resolution. To minimise the noise in bird community data associated with stochastic annual variability in environmental conditions, we selected, for each sampling point and each species, the average population count across three adjacent years (2000, 2001, 2002; 2015, 2016, 2017)
Data exclusions	For this study we focused solely on routes in the USA, as most Mexican and Canadian routes are currently still being set up, therefore data in these regions are spatially and temporally sparse. Prior to analysis, we filtered the BBS dataset by removing routes that had incomplete survey lengths (less than 50 point count stops, indicated by the RouteTypeDetailID field value being less than 2 in the extracted BBS dataset), routes that were surveyed under adverse weather conditions such as high wind and rain (as indicated by the Run Protocol ID field being equal to 1), which could affect bird occurrence and detectability. We also removed segments 2 and 4 from our analyses, thus considering only segments 1-3-5, to minimise spatial autocorrelation.
Reproducibility	Since this is a modelling study using freely available data, and we made our code available to the community, the study can be reproduced by anybody.
Randomization	Sampling was not random as data collection depends on volunteers, and it's therefore dependent on population density. We have repeated our analyses with a subselection of the data to have a more equally spatially-distributed dataset, but this did not change the results.
Blinding	We have used all bird data from the USA irrespective of who collected it and where it was collected.

Did the study involve field work? ☐ Yes ☒ No

Reporting for specific materials, systems and methods

We require information from authors about some types of materials, experimental systems and methods used in many studies. Here, indicate whether each material, system or method listed is relevant to your study. If you are not sure if a list item applies to your research, read the appropriate section before selecting a response.

Materials & experimental systems

n/a	Involved in the study
☒	☐ Antibodies
☒	☐ Eukaryotic cell lines
☒	☐ Palaeontology and archaeology
☒	☐ Animals and other organisms
☒	☐ Human research participants
☒	☐ Clinical data
☒	☐ Dual use research of concern

Methods

n/a	Involved in the study
☒	☐ ChIP-seq
☒	☐ Flow cytometry
☒	☐ MRI-based neuroimaging
